# Supplementary material for: Are carbon emissions trading and green financial instruments synergistic? -Comprehensive quantitative research based on content analysis
Source: PLoS One. 2024 Mar 7;19(3):e0298601. doi: 10.1371/journal.pone.0298601 (PMC10919601; doi:10.1371/journal.pone.0298601)
Supplement: S1 Data — (ZIP) [file pone.0298601.s001.zip › Data/地级市Z_F工作报告.zip!/─■╧─/╓╨╬└╩╨/└┤╫╘╣1⁄2╓┌║┼í╛┬φ┐╦╩2╛▌═°í┐_2017.docx]

各位代表：

现在，我代表市第三届人民政府，向大会作工作报告，请予审议，并请政协委员和列席人员提出意见。

本届政府工作回顾

2013年以来，在自治区党委、政府和市委的坚强领导下，在市人大、政协的监督支持下，市第三届人民政府认真学习贯彻习近平总书记系列重要讲话精神，主动适应经济发展新常态，积极应对经济下行压力和各种困难挑战，统筹推进稳增长、促改革、调结构、惠民生等各项工作，较好完成了任期目标任务，为全面建成小康社会打下了坚实基础。

经济总量稳步提升。2016年，全市预计完成地区生产总值340亿元，是2012年的1.4倍，年均增长7.3%，人均生产总值达29620元，净增6841元；固定资产投资370亿元，累计达到1372亿元，年均增长10.6%；规模以上工业增加值95亿元，是2012年的1.4倍，年均增长7.4%；地方公共财政预算收入23.1亿元，是2012年的1.6倍，年均增长11.8%；社会消费品零售总额65.8亿元，是2012年的1.5倍，年均增长10.6%；城镇、农村常住居民人均可支配收入分别达到23290元和8640元，比2012年增加6677元和2619元，年均增长8.8%和9.5%。

结构调整取得突破。紫光天化蛋氨酸、今飞轮毂、华创风机制造等一批项目建成投产，天元锰业完成战略重组，美利纸业成功转型。新能源装机容量3.6GW，占全区的30.4％，电解铝达到31万吨。建成亚马逊AWS、云创公司2个新一代绿色数据中心和4×100G云计算信息高速公路，使中卫乃至宁夏成为国家网络骨干节点，数据中心服务器达到3.5万台，吸引奇虎360、浪潮等140家互联网知名企业落户中卫。中卫云创数据中心被确定为首批国家绿色数据中心试点。中关村中卫园建成投用，近期吸引6100余各界人士参观考察。初步形成了以金属锰、铝基复合材料等为代表的新材料，以光伏风电为代表的新能源，以云计算、云制造、云应用为代表的新经济产业发展布局。坚持“一特三高”，打造硒砂瓜品牌核心保护区40万亩，“中宁枸杞”品牌价值达23.4亿元。建成一批农产品加工园，万齐等4家企业在新三板挂牌。粮食产量实现“十二连增”。新培育市级以上农业龙头企业150家，农业合作社1360家，宁夏红、夏华、天瑞等品牌享誉全国。加快发展现代服务业，建成旅游新镇、沙坡头水镇等一批景区景点，全国全域旅游推进会在中卫召开，中卫被列入国家首批全域旅游示范市创建单位。2016年，预计旅游人次、旅游收入分别达到550万人次和42.3亿元，均比2012年实现翻番。充分发挥中卫交通区位优势，建成干武铁路增建二线工程，扩建沙坡头机场停机坪，建设中国物流中卫物流园等一批现代物流园区，中阿国际货运班列常态化运营。黑海高速正式通车，吴忠至中卫城际铁路开工建设。四季鲜、全民创业城等商贸综合体建成运营。改造提升农贸市场28家，建设农家店800余家。全市进出口贸易额由2012年的9.3亿元增加到2016年的19.2亿元，增长1.1倍。

城乡面貌显著变化。积极开展空间规划“多规合一”改革试点工作。建成“大河之舞”等一批市民休闲公园，完成高庙历史文化街区改造，新改建城市道路94公里，新建各类保障性住房540万平方米，改造老旧小区143个、棚户区3.7万套。市区建成区面积增加到32平方公里，城镇化率41%，市辖区达56%。海原大县城建设和沙坡头区、中宁县旧城改造取得明显成效。建成美丽小城镇15个、美丽村庄115个，改造农村危房4.2万户。改扩建中卫、中宁黄河大桥。建成农村客运站349个，全市行政村覆盖率达76%。全国城市环卫保洁工作现场会在中卫召开，“以克论净”城市保洁模式全国推广，先后有387批5322人次到中卫考察学习。中卫跻身“国家园林城市”。

生态环境明显改善。出台《关于落实绿色发展理念，加快美丽中卫建设的实施意见》，实施腾格里沙漠东南缘防沙治沙、黄河卫宁城市过境段生态综合治理及城区、美丽村庄绿化提升项目，加大退耕还林、移民迁出区生态修复力度，完成造林封育32.5万亩、生态防护林资源达218.8万亩。严守耕地红线，全市实际耕地保有量达393万亩。城市绿地率和绿化覆盖率分别达32.7%和38.5%。大力推进节能减排，空气质量优良天数比率达80%以上，主要污染物排放量低于自治区控制指标。环保部挂牌督办问题摘牌，得到中央第八环境保护督察组及全国人大执法检查组、全国政协调研组的肯定。

改革开放深入推进。创新设立海兴开发区，沙坡头区以市辖区正式运转。成立市金融工作局，市属国有企业“1+X”归并重组，设立产业发展、扶贫担保等各类基金50亿元，吸引社会资本组建助贷公司、担保公司、小贷公司，有效缓解了发展资金短缺问题。深化行政审批改革，推行商事登记制度，公布责任、权力和公共便民服务事项3个清单，建立“51311”行政审批体系，在中卫工业园区试点推行行政审批告知承诺制，取消所有市本级行政收费。推进中卫、中宁工业园区直供电改革，重点用能企业天然气直供改革，天然气交易8550万立方米。农村土地承包经营权确权登记颁证工作通过自治区评估验收。沙坡头区、中宁县农村产权流转服务中心运营。完成党政机关公务用车改革。出台《加快推进开放中卫建设的实施意见》。与31个毗邻城市建立旅游协同发展机制，同甘肃白银、内蒙古阿盟等周边地区建立全面战略合作关系，宁夏大学亚马逊云计算学院列入第六轮中美人文交流成果。举办“民营企业中卫行”活动，签约项目29个，金额288.8亿元。累计实施招商引资项目251个，到位资金920亿元，是2012年的1.4倍。

社会事业快速发展。实施市级以上科技项目685个，申请专利1075件，授权440件。新建高新技术园区2个，中国人民大学西部人才培训基地、兰州大学中卫高新技术研究院挂牌成立。新建中卫九小等31所学校（幼儿园），新建、改扩建校舍64万平方米，沙坡头区、中宁县义务教育基本均衡发展县（区）创建工作通过国家评估认定。建成宁夏大学中卫校区，填补了中卫高等教育空白。建成中卫体育休闲公园等一批公共文化设施，乡镇文化综合站“公建民营公助”模式全面推开。举办全国大漠健身运动大赛等自治区级以上体育赛事20场次。大力开展群众性精神文明创建活动，中卫市先后荣获“全国未成年人思想道德建设工作先进城市”和“自治区文明城市”。建成市人民医院医疗中心、自治区人民医院宁南分院，完成海原县人民医院迁建，新建标准化村卫生室304所。成功创建自治区卫生城市。计划生育政策全面落实。推进“平安中卫”建设，建立城市大巡防体系，命案和八类主要刑事案件发案率分别下降45.4%、12.2%。完善“1+X”信访工作机制，化解疑难案件263件。率先在全区推行安全生产第三方监管服务。加强食品药品监管。启动实施民族团结进步、“一争三创五推进”活动，依法加强宗教事务管理。加强国防动员和后备力量建设，积极拓展军民融合领域。中卫被评为全国“双拥模范城”。

办结一批民生实事。承诺并办结民生实事52件。财政投入民生16.2亿元，是2012年的5.4倍。创新“有土”“离土”两条脱贫路径，51个贫困村脱贫销号，10.6万贫困人口精准脱贫。新建、续建生态移民住房9218套，搬迁安置移民3.9万人，全面完成“十二五”移民搬迁安置任务。华润集团“基础母牛银行”等精准扶贫模式得到中央肯定。中卫市第二水厂建成投运。脱贫攻坚水源工程顺利推进，中南部城乡饮水安全海原县连通工程全线通水，全市农村饮水安全问题基本解决。创新建立“四化一满意”服务群众工作机制。财政贴息3858万元，为6342名创业者提供小额担保贷款6.4亿元，创业带动就业2.2万人。建成幸福院156个、敬老院14个、老饭桌62个，投入运营养老床位2235张，“五保”集中供养率达24%。建成公共租赁住房1.5万套，1.2万户贫困家庭圆了“安居梦”。累计为51.9万名城乡低保对象发放保障金13.4亿元。一次性解决了1.2万套3421户群众的逾期安置问题，4.5万名被征地农民全部纳入养老保险，在全区率先将市本级1万余名事业单位人员全部纳入医疗补助范围。中卫市被人社部确定为社会保障卡综合应用全国首批示范基地。

政府效能不断提升。深入开展党的群众路线教育实践活动、“三严三实”专题教育和“两学一做”学习教育。组织开展了以掌握“三基本”为主要内容的“二次创业”大讨论活动，深化了市情认识，推进了思想解放。圆满完成“六五”普法任务，启动“七五”普法工作，扎实推进法治政府建设。累计办理区、市人大代表建议208件、政协委员提案801件，办理答复率均为100%。出台《中卫市重大行政决策规则》，召开市政府常务会议90次，决定重大事项840个。组建中卫仲裁委员会，成立行政复议委员会。推行网上办公，全面公开政务信息，实行市长信箱、政务微博、媒体问政综合联动，市长信箱受理群众来信来电1.3万件，办结答复率100%。严格执行中央八项规定和区、市相关规定，清退办公用房5.4万平方米，大幅精简会议文件，“三公”经费年均下降17.3％。出台《公共资源交易管理办法》，政府投资项目全部采用合理造价区间随机抽取方式确定中介服务机构，节约资金12.2亿元。全面落实从严治党“两个责任”，严肃查处一批违法违纪案件，形成了风清气正的政治生态。

统计、粮食、供销、档案、地方志、妇女儿童、外事侨务、消防、气象、防震减灾等各项工作均取得新成绩。

各位代表，回顾本届政府工作，我们经受了前所未有的经济下行压力，办成了一批增进人民福祉和事关中卫长远发展的大事、难事，一个年轻而富有魅力、开放而彰显活力、靓丽而充满实力的中卫屹立在祖国西部！成绩归功于自治区党委、政府和市委的正确领导，人大、政协和社会各界的鼎力支持，历届政府打下的良好基础和全市人民的务实苦干。在此，我代表市第三届人民政府，向辛勤耕耘在各条战线的广大干部群众，向给予政府工作大力支持的人大代表、政协委员、各民主党派、工商联和各界人士，向中央、区属驻卫单位、驻卫部队、武警官兵致以崇高的敬意!向所有关心支持中卫发展的各界朋友表示衷心的感谢！

百舸争流，奋楫者先；千帆竞渡，勇进者胜。各位代表，回顾本届政府工作，我们的前进征程步履坚实，我们的发展成果来之不易，我们的创业体会弥足珍贵。在看到成绩的同时，我们也清醒地认识到，中卫建市晚、底子薄、经济总量小、人均水平低、贫困程度深仍是我市最基本的市情，从“量的积累”转向“质的跃升”还有差距。主要表现在：经济下行压力持续加大，新产业新模式还在成长，新的发展动能还在孕育，新旧动力转换“青黄不接”；山川、城乡、区域发展不平衡，城市化水平低，脱贫攻坚任务艰巨；财政收入总量小与公共服务、民生保障等刚性支出增加的矛盾依然突出；一些干部思想不够解放，尤其是少数干部庸政、懒政、怠政等问题有待进一步解决。

今后五年的目标任务

今后五年，是中卫加快转型升级、决胜脱贫攻坚、全面建成小康社会的关键时期。市第四次党代会提出了“一个提前翻番、两个重大突破、三个明显缩小、五个全面提升”的目标，绘就了发展蓝图，指明了前进方向。当前，中卫正处于新旧动能转换的“弯道”上，我们要紧紧抓住国家“一带一路”倡议、脱贫攻坚、西部开发等重大战略，推进供给侧结构性改革和大众创业、万众创新，用足用活宁夏“内陆开放型经济试验区”“全域旅游示范区”等“金字招牌”，准确把握新常态下中卫发展的坐标定位和阶段特征，有效应对改革发展中的各种困难和挑战，牢固树立“川区争上游、山区争先进”的“双争意识”，以新动能、新引擎、新路径、新活力“四新”要求推进经济转型升级，实现增速换挡、弯道超车，在新的历史征程中赢得主动、赢得优势、赢得未来。

今后五年政府工作的总体要求是：高举中国特色社会主义伟大旗帜，以邓小平理论、“三个代表”重要思想、科学发展观为指导，全面贯彻党的十八大和十八届历次全会精神及自治区党委、政府和市委决策部署，深入贯彻习近平总书记系列重要讲话精神和治国理政新理念新思想新战略，按照市第四次党代会的部署要求，牢牢把握“转型追赶、弯道超车”这条主线，大力实施山川协同发展、经济转型发展“两大战略”，更加注重开放引领，更加注重创新驱动，更加注重生态优先，更加注重富民共享，全面推进经济建设、政治建设、文化建设、社会建设、生态文明建设和党的建设，坚决打赢脱贫攻坚战，努力实现经济繁荣、民族团结、环境优美、人民富裕，奋力开创开放富裕和谐美丽中卫新局面，确保与全区全国同步建成全面小康社会。

今后五年的发展目标是：经济保持中高速增长，地区生产总值年均增长8%左右，地方公共财政预算收入与经济增长同步，城镇、农村常住居民人均可支配收入年均分别增长9%和10%左右，确保实现“一个提前翻番、两个重大突破、三个明显缩小、五个全面提升”目标。

“一个提前翻番”，即对照2020年全面建成小康社会目标，地区生产总值提前两年在2010年基础上实现翻番，到2021年突破550亿元大关。

“两个重大突破”，即西线扶贫引水、城际高铁等重大基础设施项目全面建成，卫宁相向发展和海原大县城建设加快推进，带动山川协同发展取得重大突破；云计算和大数据、全域旅游、现代物流等特色产业发展壮大，带动经济转型取得重大突破。

“三个明显缩小”，即城镇、农村常住居民人均可支配收入和城镇化率三项指标与全区平均水平的差距明显缩小。

“五个全面提升”，即全民思想道德素质全面提升，基本公共服务均等化水平全面提升，社会治理能力全面提升，生态环境质量全面提升，政府推动经济社会发展的能力全面提升。

重点抓好以下五个方面工作：

一、以改革创新为动力，加快建设开放中卫

向改革要动能。深化“放管服”改革，设立统一的行政审批机构，全面实行告知承诺制，将分散在部门的所有审批职责集中，实行一个部门管审批，推动行政审批全面迈入“单点单部门”方式，实现网上审批、网上注册、网上办事全覆盖。建成“政务云”，大力推行“互联网+政务服务”，实现市直所有审批部门业务数据联通，与国家部门、区级系统对接，推动政府信息共享。深化农村综合改革，完成供销社、国有林场改革，推进土地所有权、承包权、经营权分置管理，推进农村集体资产确权到户和股份合作制改革，激发农村活力。深化投融资改革，积极吸引全国性股份制商业银行、证券公司、保险公司设立分支机构，进一步完善金融服务体系。做强“1+N”国有投融资平台，不断提升投资质量和效益。培育新三板挂牌企业10家、后备上市企业10家。深化要素保障改革，争取企业气价优惠、电价下降，实现水资源合理配置。

向开放要活力。深化区域合作，加强与银川、兰州、西安等周边区域中心城市对接合作，促进产业协作配套、基础设施共建共享，构建“中卫1小时经济圈”。密切与北上广深等沿江沿海地区交流合作，高起点承接产业转移。打造开放平台，主动融入国家“一带一路”发展战略，依托宁夏内陆开放型经济试验区和中阿博览会，办好中阿博览会中国枸杞论坛暨中宁枸杞文化节，将云计算设立为中阿博览会常设分论坛。加强与迪拜多种商品交易中心（DMCC）、印度国家信息学院（NIIT）等合作，促进贸易流通。发挥“中阿号”国际货运班列作用，推进陆路口岸保税点建设，建成迎水桥铁路编组站海关监管场所，争取设立镇罗公铁物流中心保税仓库。加强与汉新欧、天津港等合作，实现中宁陆路口岸封关运营，建成银川保税区的配套区。办好环湖自行车、沙排、轮滑等国际赛事，促进对外合作交流。提升开放实效，紧盯世界500强、中国500强企业，突出以商招商、专业招商、委托招商，在主导产业链延伸和耦合发展上谋划引进项目，推动招商引资工作由“招商”向“选商”转变，由引资金向“引特色产业链”转变，打造优质、高效、便捷的开放环境。2021年，培育引进投资百亿元龙头企业4家，招商引资到位资金突破1600亿元，进出口总额年均增长8%以上。

向创新要效益。深入实施创新驱动发展战略，加强重点领域、重点行业科研攻关和科技成果转化应用，争创国家高新技术产业开发区。围绕产业转型升级，强化科技创新体系和基地建设，构建“1+2+3”科技创新体系。高标准建设中卫国家农业科技园区、沙坡头区国家现代农业示范园区、中宁枸杞科技产业园，推进科技研发与应用。实施企业技术中心建设工程，推动天元锰业、锦宁铝镁等企业创建国家级技术创新中心和工程技术研发中心，提高企业核心竞争力。紧扣产业链和创新链，创新人才政策，完善人才服务机制，加强海外人才合作，大力引进培养高层次人才。2021年，全社会研究与试验发展经费支出占地区生产总值比例达2%左右，高新技术企业达10家以上，科技进步对经济增长的贡献率达55%。

二、以转型升级为突破，加快建设富裕中卫

做大做强新型工业。实施中国制造2025宁夏行动纲要，推动支柱产业倍增计划，建设自治区新型工业化示范基地。推动产业集聚发展，大力发展“三新”产业，重点发展铝板带箔、钒钛合金、镍钛合金等产业，壮大原铝—型材—铝板带箔—轮毂制造链条，推进新材料产业集群化；重点发展协鑫高效单晶硅、协佳电池组件、隆基硅多晶硅等产业，推进新能源产业高端化；重点发展云计算配套电器设备制造、机架生产等电子信息产业，推进新经济规模化，打造“一个千亿元、四个百亿元”产业集群。实施工业强基工程，提升传统产业装备水平，抓好天元锰业、宁夏钢铁等企业扩规改造，推进钢铁、铁合金、电石企业循环改造，推广余热余气利用，促进生产、流通、消费过程减量化、资源化、再利用。完善精细化工产业链，构建“上下游密切关联、资源能源互供互享”循环经济产业体系。引导产能过剩企业实施兼并重组，处置“僵尸企业”，化解过剩产能，淘汰落后产能。打造低成本工业园区，积极发展分布式新能源，建设园区微电网，开展直供电交易，探索园区节电模式。实施园区供水、供电、供气、供热和集污管网改造，加快建设园区集中供热站和工业管廊，降低企业生产要素成本，实现园区基础设施共建共享，提升园区公共服务能力，增强园区承载力。推动中卫、中宁工业园区一体化发展，加快中卫、中宁工业园区铁路专用线建设，推进国铁、地方铁路融合直通，构筑量价联动的物流体系，切实降低物流成本。2021年，全市工业增加值达165亿元，工业对经济增长贡献率达38%。

做精做特现代农业。优化“四区七带”产业布局，紧盯“1+5”优势特色产业，重点打造120万亩优质粮食产业带、50万亩“原产地”枸杞产业带、40万亩蔬菜产业带、70万亩优质经果林产业带、60万亩马铃薯产业带、100万亩牧草+200万“羊单位”草畜产业带和100万亩硒砂瓜产业带，特色产业占比达87％。推进枸杞产业提质增效，提高特色化、品牌化、精品化发展水平，依托电子商务孵化中心、枸杞电子交易中心等平台，在各大中城市实施“百城千店”计划，拓展营销网络，让全国人民都能吃上中宁枸杞，硒砂瓜、马铃薯、供港蔬菜等特色农产品走出国门。以枸杞精深加工、优质粮食和清真牛羊肉加工、乳制品生产为重点，培育壮大中宁新水农产品加工园，规划建设沙坡头、海原农产品加工园，扶持万齐、早康、夏华等农业龙头企业做大做强。深化义乌、中卫两地战略合作，搭建农产品产销平台。严把农产品品质和安全关口，创建无公害农产品、绿色食品地理标志380个，加强农产品检测和追溯体系建设。2021年，全市农产品加工企业达1300家，农产品加工转化率达70%以上。强化农业经营主体和服务支撑体系培育，提高土地规模化经营水平，新型农业经营主体达1.5万家以上，农业社会化服务机构达30家，农村土地流转率和土地规模经营率均达40%以上。

做活做靓现代服务业。围绕打造全域旅游城，全面落实《中卫市全域旅游发展行动规划（2016—2018年）》，加快实施城市综合提质、基础设施完善、品牌战略营销等八大工程，构建“一核、两翼、三带、四区、六线、多节点”全域旅游新格局。坚持重点景区提质扩容，一般景区“创A升级”，实施沙坡头“东扩、西延、南跨、北拓”工程，增强沙坡头景区的聚合力。深化与港中旅合作，力争港中旅“十三五”期间在中卫投资20亿元左右，使沙坡头旅游景区成为集观光体验、休闲度假为一体的复合型景区。加快推进腾格里湿地、沙坡头水镇、海原非物质文化遗产传承基地等景点景区建设，完善中宁、海原旅游吸引物系统。实施黄河卫宁城市段治理工程，深度开发黄河资源，打造旅游新业态。推动沙坡头景区和腾格里湿地跻身国家级度假区行列。大力推进“旅游+”和“互联网+”，促进旅游与新型工业、特色农业、文化体育等融合发展，实现倍增效应。争取沙坡头文化旅游产业示范区实体运作，完善“1+4+N”全域旅游管理模式，打造“快旅慢游”交通体系，为游客提供各环节、全方位的服务。推动星级景区、酒店和乡村旅游示范点免费WIFI覆盖，支持景区、酒店、旅行社等旅游企业发展网上预订、在线支付等电子商务，推动品牌景区和品牌线路率先实现旅游服务智能化。全面创建全域旅游示范市。2021年，全市力争五A级景区达到2家，四A级景区达到3家，三A级景区达到5家，接待游客1240万人次，旅游收入达到136亿元。围绕打造国际云天城，加快亚马逊AWS、云创二期、中国移动等数据中心建设，引进美国ZT、浪潮等企业在中卫建设云计算配套生产基地，引进集成电路板制作等电子工业产业落地中卫，逐步形成云存储设备、桌面云终端等制造特色产业集群。依托中卫西部云基地，构建“一带一路”区域数据中心和云计算平台，促进与阿拉伯国家空间信息产业合作。争取国家工信部、安全部、中国气象局等部门数据中心落户中卫，建设国家战略安全数据灾备中心，实现多级数据融合和大数据共享。贯彻大数据军民融合发展战略，以云天产业园为载体，建设天地一体化信息网络系统地基信息港，积极推进军民两用飞艇基地、火星探测科普体验基地、北斗导航卫星数据应用产业基地、国防教育及武器装备科研试验基地建设。推进智慧中卫建设，深化政务、社保等“8+N”覆盖广度和应用深度，为公众提供跨部门、一站式便捷信息服务。建立统一的大数据服务平台，推进政府信息公开、资源共享和数据开放。对标美国凤凰城，抢抓大云西移机遇，盯紧“一个枢纽、三个基地”发展目标，创新“前店后厂”区域合作模式，着力在中卫建设国家大数据综合试验区，先行先试“一体化国家大数据中心”示范区建设。围绕打造交通枢纽城，抓住国家实施“一带一路”和向西开放机遇，加强与新丝路、汉新欧合作，加强与天津、青岛、连云港、满洲里、阿拉山口、霍尔果斯等沿海港口、沿边口岸、铁路枢纽和航空港的贸易对接，实现国内东进西出的货物在中卫集结，推动货物通关、商检一体化运作，打造承东启西的宁夏国际陆港货物集散地。积极发展公铁物流、公海物流、多式联运等物流形式，建成乌玛高速中卫段等高速公路、吴忠至中卫城际铁路、中卫至兰州客运专线、中卫高铁站跨黄河大桥。推进宝中铁路平凉至中卫段扩能改造等项目建设。争取开行中欧班列，扩建沙坡头机场，加密中卫至北京、上海、西安航班，使公路、铁路、航空立体交通网络更加完善、更加便捷，打造亚欧大陆物流中转基地。2021年，建成五横两纵高速公路网、通往国内外连接欧亚大陆桥的“米”字型铁路网。加快镇罗公铁物流园、海原物流园等物流园区建设，争取设立上海石油天然气中卫交易中心和宁东物流园区中卫仓储区，让更多的人流、物流、信息流汇聚中卫。引进龙头企业发展第三方物流，稳步发展冷链物流，积极发展电商物流，整合快递营业点、农村电商村级服务站等资源，推进“快递+互联网”“快递+电商”协同发展。

三、以民生改善为目的，加快建设和谐中卫

坚决打赢脱贫攻坚战。因地制宜推进“有土”脱贫，全面落实“离土脱贫23条”政策，实施“产业发展、劳务输出、易地搬迁、教育脱贫、社会兜底”五项脱贫行动计划，按照宜农则农、宜林则林、宜牧则牧、宜商则商的原则，培育发展枸杞、草畜、马铃薯、硒砂瓜等特色产业和休闲农业、光伏产业、农村电商、乡村旅游等新业态，确保有劳动能力的贫困户每户有1-2个稳定增收的产业项目，实现产业脱贫4.17万人。依托劳务移民、插花移民、县内移民等，实现易地搬迁脱贫1.55万人。全面落实国家教育扶贫各项政策，对建档立卡贫困户大学生、研究生每人每学年再资助5000元，支持贫困家庭子女更多地依靠教育走出去，实现教育脱贫1.6万人。以汽车运输、清真餐饮、剪纸刺绣等为重点，加大技能培训，未升学初高中毕业生全部接受职业教育，鼓励自主创业、进城务工，引导劳务输出脱贫2.29万人。完善农村最低生活保障制度，实现农村扶贫线和低保线“两线合一”，将所有符合条件的贫困群众纳入低保范围，推进大病医疗保险全覆盖，实现政策性保障兜底脱贫2.99万人。加大金融扶贫力度，全面开展精准扶贫“脱贫保”保险业务，探索建立财政扶贫资金资产收益分配机制，从根本上解决贫困群众贷款难问题。配套完善水、电、路、通讯等基础设施，重点抓好宁夏中部干旱带西线引水工程，建设沙坡头区至海原、海原至平川快速通道。启动实施城乡一体化供水工程，进一步提高农村饮用水安全保障水平。提前完成“十三五”易地搬迁安置任务。实行基本养老、医疗保险补贴，提高贫困人口医疗救助和报销比例，切实解决因病返贫等问题。出台贫困群众提前脱贫激励政策，引导贫困群众自力更生、艰苦奋斗，主动脱贫。深化闽宁对口扶贫协作，创新社会帮扶机制。落实脱贫攻坚责任制，充分发挥机关、干部帮扶作用，形成全社会共同参与的大扶贫格局。

全面发展社会事业。加大教育基础设施建设力度，新建、改建中小学校（幼儿园）140所。实施学前教育办学能力提升工程，加大政府购买学前教育服务力度，鼓励引导普惠性民办幼儿园发展，实现有需求行政村幼儿园全覆盖。实施义务教育均衡发展攻坚计划，缩小区域、城乡、校际差距，海原通过国家义务教育均衡发展评估认定，中卫迈入全国义务教育均衡发展先进市（县）行列。2021年，学前3年毛入园率达88%以上，小学6年巩固率达97.5%，初中3年巩固率达98%，高中阶段毛入学率达98.5%。加快发展现代职业教育，建设国家级现代职业技能公共实训中心，打造西部领先、机制一流的职业教育高地。支持办好宁夏大学中卫校区。支持和规范民办教育健康发展。扎实推进“名师名校名校长建设工程”，稳步提高教师待遇。深入推进教育惠民工程，实行贫困家庭学生学前两年全免费、普通高中家庭困难学生全免费、中等职业学校学生全免费、贫困大学生生源地信用助学贷款全覆盖、家庭经济困难学生资助全覆盖。大力实施医疗设施保障、医护人员能力“两项提升计划”。迁建市中医医院，实现标准化村卫生室和社区卫生服务中心全覆盖。深化与北京、上海等区内外知名医院合作，加强远程医疗会诊，推动医护人员培训交流，提升医疗卫生服务能力。2021年，全市常住人口每千人医疗卫生机构床位数达到4.8张，县域内就诊率达85%左右，基本建成覆盖城乡的基层医疗卫生服务体系。建设市级远程医疗会诊平台并连通所有基层医疗卫生机构。持续推进城市公立医院综合改革，市县二级以上医院全部实行药品零差率销售。加快推进异地就医结算，实现医疗、医保、医药“三医”联动。坚持计划生育基本国策，促进人口素质提高和长期均衡发展。推进健康城市健康村镇建设，深入开展爱国卫生运动，争创国家卫生城市。进一步完善市、县、乡、村四级公共文化服务设施，建设3个县级青少年文化宫，实现文体设施社区全覆盖、村级综合文化服务中心行政村全覆盖、标准化乡镇综合文化站全覆盖。实施文化惠民工程，深入开展文化“六进”活动，引导各类文化资源向基层倾斜。建立文化消费补贴机制，向低收入群众发放文化消费卡，为城乡居民提供“普惠式”文化消费。依托中卫云天产业园，加快培育文化创意、数字动漫、影视创作等新型文化业态。扶持大麦地阳光文化产业园、中宁枸杞文化产业园、海原回族文化创意产业园等文化产业示范基地，推进文化、旅游、城市建设融合发展。深入开展群众性精神文明创建活动，争创全国文明城市。

做好就业创业和社会保障工作。统筹做好高校毕业生、城镇就业困难人员、退役军人、进城务工人员、零就业家庭等就业工作。健全劳动合同和劳动争议仲裁制度，保护劳动者合法权益，构建和谐劳动关系。推动大众创业、万众创新，加快发展“互联网+”创业网络体系，搭建开放式的众创、众包、众扶、众筹平台。2021年，力争实现城镇新增就业4.2万人，城镇登记失业率控制在4.5%以内。实施全民参保计划，稳步提高城乡居民基本养老金标准，稳妥实施被征地农民参加养老保险工作，完善机关事业单位养老保险制度，整合社会保险经办资源，实现“五险合一”经办管理，构建城乡一体社会保险体系。实施社会保障扩面提标工程，织好基本民生安全保障网。健全社会保障待遇标准与物价、收入相协调的联动机制，完善住房保障制度，启动实施干部职工住房补贴制度，稳步提高社会保障水平。建设医养结合养老产业园区。建立健全居家养老和社区养老服务网络，做好困境儿童、农村留守儿童、妇女老人等关爱保护工作，提高残疾人福利补贴标准，完善城乡社会救助和社会福利体系。2021年，全市法定人口基本社会保险、困难群众救助兜底、困难失能失智老人救助补贴、养老服务实现全覆盖。

加强和创新社会治理。推进社会组织、社会工作和社区单位“三社联动”，努力建设更高水平的平安中卫。创新社会治安综合治理，加快建设立体化、信息化社会治安防控体系。完善突发公共事件应急管理体系，加强安全生产工作，提高重点行业领域安全治理能力，坚决防止重特大安全生产事故发生。推进食品药品安全治理体系建设。建立健全有机衔接、协调联动、高效便捷的矛盾纠纷多元化解机制，全面落实信访工作责任制，依法分类处理信访诉求，维护群众合法权益。全面落实党的民族政策，深入开展民族团结进步创建活动，争创全国民族团结进步示范市。依法管理宗教事务，严格清真标识的审批、使用和管理，严禁公共场所和世俗生活宗教化，巩固民族和睦、宗教和顺、社会和谐的良好局面。

四、以统筹协调为关键，加快建设美丽中卫

落实空间规划。扎实做好“多规合一”试点改革，科学界定主体功能区，严控建设用地规模，严守基本农田红线，确保生态功能不降低、面积不减少、性质不改变。按照“产业连城、生态连城、水系连城、交通连城”思路，规划建设沙坡头区至中宁快速通道，推进沙坡头区和中宁县城相向发展。准确把握城市生产、生活、生态空间的内在联系，注重城市功能布局，打响“滨河沙坡头、杞乡中宁、生态海原”品牌，打造宜居、宜业、宜游新中卫。

打造美丽城市。坚持新城、老城联动发展，持续推进美丽城市“九大工程”，争取实施城市地下综合管廊工程。完善沙坡头水镇、旅游新镇、高铁站核心区配套，加快推动产业项目集聚，全面提升综合承载能力。实施市区道路畅通工程，新建市政道路89公里。继续推进既有建筑节能改造，基本完成城中村、城边村、重点集镇棚户区和农村危房改造。扎实推进绿地公园、立体停车场、城市充电桩等项目建设，完善供水、供热、供气等老旧管网改造，有效解决交通拥堵、停车难等城市病。推进农民工市民化，继续深化户籍、养老等制度改革，促进有能力在城镇就业和生活的农业转移人口举家进城落户，并与城镇居民享有同等权利和义务，实现基本公共服务常住人口全覆盖。2021年，全市城镇化率达52%。

建设美丽乡村。建设美丽村庄72个，改造危房1.4万户，农村砖房率达91%。配套完善供水、供热、通讯、道路、垃圾处理等设施。巩固“5个3”乡村环境卫生长效机制。统筹生态移民迁入地村庄建设，实施农村电网改造升级工程，鼓励发展农村清洁能源。完善农村公路网络，实现所有行政村通客车。实施新农村现代物流网络工程。推进农村生态示范区建设。2021年，全市所有乡镇、村庄达到美丽宜居乡村标准。

保护美丽环境。强化北部沙区林草建设管护，建设市民休闲森林公园，提升中卫、中宁等工业园区绿化层次，推进移民迁出区生态修复，巩固扩大生态经济林面积。切实保护生态资源，坚守“生态红线”，落实生态补偿机制和责任追究制度，加强林地、林木资源管理，严厉查处涉林违法行为。2021年，全市规划新增造林面积120万亩，森林覆盖率达14%。深入实施蓝天、绿水、净土、工业污染治理等行动计划，精心呵护好中卫的绿水青山。突出整治工业污染，加大“三废”治理力度，严格落实国家区域限批、行业限批等规定，全面落实重点企业在线监测、IC卡管理等措施，严惩偷排超排，坚决关紧污染“排放口”。加大燃煤锅炉、工业烟粉尘、机动车尾气、城市扬尘治理力度，加快推进城市集中供热，完成沙坡头区、中宁县城市燃煤供热锅炉替代工作，海原县天然气实现联通。全部淘汰黄标车。依法保护饮用水源地，加强地下水污染防控，确保百姓用上放心水。加强城市污水处理厂技术改造，推进城镇污水集中处理。强化农业面源污染治理，提高农作物秸秆、农用残膜利用率。2021年，全市污水处理率达95%以上。

五、以职能转变为抓手，切实加强政府自身建设

建设法治政府。坚决服从市委领导，自觉接受市人大及其常委会、市政协和社会各界的监督，认真落实市人大及其常委会的决议决定，认真吸收市政协及社会各界的意见建议，改进人大代表建议和政协提案办理方式。全面贯彻《法治政府建设实施纲要》，深入开展“七五”普法，全面推进依法治市，推动全社会尊法、学法、守法、用法。完善依法行政制度体系，坚持公众参与、专家论证、风险评估、合法性审查和集体讨论决定，推进政府决策民主化、科学化和法治化。2020年，基本建成职能科学、权责法定、执法严明、阳光透明、廉洁高效、守法诚信的法治政府。

建设责任政府。全面推行政府绩效管理，坚决整治不作为、慢作为、怕担当、不负责等庸政懒政怠政问题。说了算、定了干、马上办，加强督促检查，切实解决重安排、轻落实，有令不行、有禁不止，推诿扯皮、敷衍塞责等问题。坚持抓严抓紧抓细抓实，走在前列、干在实处，恪尽职责、善做善成。

建设服务政府。坚持问政于民、问需于民、问计于民，努力使政府作出的决策部署、出台的政策措施更加符合人民群众期待，政府工作更加切合人民群众需求，公共资源更多向民生倾斜。脚踏实地为群众办实事、解难事，给予困难群众更多关心和帮助。

建设阳光政府。突出抓好财政预决算、公共资源配置、重大项目建设等信息公开，做到行政决策公开、执行公开、管理公开、服务公开和结果公开，主动接受社会和舆论监督。推进政务信息服务平台建设，完善例行发布、应急发布、政策解读等机制，着力提高政务运行质量和水平。加强政府门户网站建设，不断提升传播能力，打造互联网时代政府信息公开平台、便民服务平台、政民互动平台。建立健全政务舆情收集、研判和处置机制，及时回应社会关切。

建设廉洁政府。严格执行《关于新形势下党内政治生活的若干准则》和《中国共产党党内监督条例》，切实加强政府系统党风廉洁建设，完善权力运行制约和监督机制。加强行政监察和审计监督，坚持把纪律和规矩挺在前面，把握运用监督执纪“四种形态”。加强执纪审查，严肃查处腐败案件，增强拒腐防变和抵御风险能力，构建不敢腐、不能腐、不想腐的有效机制，推进干部清正、政府清廉、政治清明。

2017年重点工作

2017年，是中卫实现转型追赶、弯道超车的关键一年。全市经济社会发展的主要预期目标是：地区生产总值增长8%左右，地方公共财政预算收入增长8%，全社会固定资产投资增长10%左右，社会消费品零售总额增长8%，城镇、农村常住居民人均可支配收入分别增长8%和9%左右。

实现上述目标，重点抓好九个方面的工作：

一、扩大有效投资，增强经济发展后劲。深入开展“项目建设年”活动，严格落实工作责任，用大项目、好项目促投资、保增长、惠民生，全年计划实施项目374个，总投资1675亿元，完成投资499亿元。开工建设工业、旅游、云计算、交通物流、农业及生态水利、城市基础设施、社会事业等重点项目60个。紧扣国家政策走向，谋划争取一批符合产业政策、支撑长远发展的重大项目，重点围绕自治区60大庆，使我市更多项目进入自治区和国家盘子，争取更多项目列入国家“十三五”规划，提前推进。大力实施“招商引资年”活动，创新招商引资方式，抢抓中阿博览会、全国工商联常委会暨民营企业助推宁夏创新发展大会在宁召开机遇，加强对接，主动沟通，通过“走出去、引进来”，确保招商引资签约资金1000亿元。

二、优化结构布局，力促工业转型升级。培育壮大“三新”产业，推动传统产业改造升级。加大协调服务，确保天元锰业四期30万吨金属锰、今飞公司500万件汽车轮毂等10个项目达产达效；加快工程进度，确保宁钢60万吨高速线材、利安隆高分子助剂等12个项目建成投产；加强项目前期，力促中化国际锂电池正极材料、协鑫晶体二期2GW高效单晶硅等10个项目开工建设。全面落实各项惠企政策，实行“一企一策”“保姆式”服务，及时协调解决企业发展中出现的困难和问题，帮助企业渡过难关。加快园区供电、供水、道路、污水处理、管网等基础设施建设，提升园区综合服务能力。严格落实工业节能降耗，着力化解过剩产能，淘汰低端落后工艺。加快科技合作和成果转化应用，培育认定国家级高新技术企业累计达10家，高技术产业增加值占规模以上工业增加值比重达10%。推进“宽带中国”示范城市建设，实现所有行政村光纤全覆盖。全市工业增加值达100亿元，同比增长5%以上。

三、转变发展方式，培育壮大特色农业。加快发展现代农业，抓好粮食、枸杞、蔬菜、苹果、硒砂瓜、草畜、马铃薯等优势特色产业。创建粮食产业攻关示范区5000亩、优质水稻示范区1万亩、玉米水肥一体化示范基地2万亩。实施经果林低产园改造1.5万亩。加快枸杞、苹果扩规提质，新增枸杞2.2万亩、苹果9500亩。开工建设江苏绿港设施标准化示范基地，新建永久性蔬菜基地1万亩。推行硒砂瓜“小产区”管理，培育万亩以上小产区5个。打造香岩集团等2个自治区级草畜示范点，加快推进中地乳业集团等万头奶牛场建设，全市肉牛、肉羊饲养量分别达33万头和260万只，存栏奶牛6.5万头。完善脱毒种薯三级繁育体系，建设2个万亩种薯繁育基地，示范推广一级种薯20万亩。引导扶持家庭农场、合作社等新型农业经营主体，新建社会化综合服务站10个。建成中卫市农产品质量安全检验检测中心。加快休闲农业发展，创建示范点3个。全市农业增加值达59亿元，同比增长5%。

四、围绕提质扩容，加快全域旅游进程。投资34亿元，重点实施沙坡头景区提质扩容、沙坡头南岸半岛、腾格里湖高墩湖酒店、中宁胜金关至黄河大桥以东沿河景观廊道、海原非物质文化遗产传承基地等项目，不断提升和完善旅游基础设施。建成智慧旅游一期项目。打造以童家园子、桂王花园等为重点的一批乡村旅游，新增乡村旅游示范点10家。完善旅游交通组织体系和旅游吸引物系统。做大做强旅游融资平台。加大与港中旅合作，督促落实投资7亿元。开发“冬日威胁”网景游戏，实现线上虚拟景区与线下美景互动。推进“十百千万”工程，完善“1+4+N”的全域旅游管理模式，重拳整治“黑导游”“黑出租”等违法行为。全市接待游客680万人次，实现旅游总收入53亿元，分别增长23.6%和23.5%。

五、抢抓战略机遇，加快壮大信息产业。实施好自治区“三号工程”，加快推进西部云基地建设，争创国家大数据综合试验区。集中力量推进亚马逊AWS、奇虎360数据中心服务器安装运营。加快中国移动、云创二期、中国联通、可信云等数据中心项目建设，率先建设全国一体化的国家大数据中心先导示范区。统筹建设云计算展示馆及配套服务中心，加快建设中卫市中小客户孵化数据中心项目，完善运营模式，促进中小企业集聚孵化。紧盯北上广深等重点区域和华为、京东、美团、IBM等重点企业，千方百计对接，力促落地。引进服务器组装、钢构生产、机房运维等云设施产业，深度挖掘数据应用，促进政务云、医疗云、环保云等云应用项目建设，打造云计算上下游全产业链。积极与中央军委和国家相关部委对接，力争将国家级卫星遥感、导航数据云平台（灾备中心）、天地一体化信息网络系统地基信息港落户西部云基地，建设军民两用飞艇基地一期工程、国防教育和武器装备科研试验基地。

六、发挥区位优势，构建现代物流体系。完善交通物流网络，开工建设沙坡头区至海原、海原至平川快速通道，扩建平凉至中卫铁路、太中银铁路，加快建设吴忠至中卫城际、中卫至兰州客运专线等铁路，疏浚中卫经中宁至青铜峡水上旅游航道。加快建设中国物流中卫物流园等6个物流园区，建设迎水桥铁路口岸，推进中宁陆路口岸封关运行。稳定开行中卫—中亚（中阿号）国际班列，吸引汉新欧等欧洲国际班列在中卫铁路口岸集结货物。加快与新疆国际陆港公司合作，争取将迎水桥编组站作为乌西编组站的前沿。加快市场培育，发展钢铁、金属锰、精细化工、建筑材料等配套物流产业，打造区域性生产资料转运中心。依托城乡物流配送、邮政配送、连锁经营等现代经营方式，构建覆盖城乡的生活性物流服务网络。加强与阿里巴巴、京东等企业对接，进一步扩大中宁电商孵化中心等规模，推动全市电子商务快速发展。

七、强化统筹协调，推进城乡一体建设。坚持新城开发与老城改造并举，改造各类管网128公里，换热站11座，新建市政道路27公里，开工建设城市停车场，提升城市亮化水平。实施棚户区改造9000套，完成安置住房1650户5000套，改造老旧小区31个。加快海原大县城及沙坡头区、中宁县旧城改造建设，建成美丽小城镇4个、美丽村庄15个。实行最严格的环境保护制度，实施大气、水、土壤污染防治行动计划，加快城区集中供热、燃煤锅炉改造，市区实现热电联产集中供热，沙坡头区、中宁县淘汰20蒸吨以下供暖和工业燃煤锅炉。严管严查扬尘污染，抓好建筑施工扬尘、渣土车、黄标车淘汰管控等工作。建成运营中卫工业园区中水厂。在所有国控、省控、市控重点企业，安装在线监测设施和智能IC卡排污监控系统，重拳打击企业偷排，实现全方位、全覆盖、无缝隙监管。实施“退耕还林、防沙治沙、兴林富民、湿地保护、绿化美化”五大生态工程，完成造林23.6万亩，治理荒漠化土地6.5万亩，补播改良天然草原95万亩。

八、推动重点改革，释放创新发展活力。继续简政放权，加快相对集中行政许可权改革。权力清单涉及的209项行政审批事项全部进驻市政务服务大厅。抓好空间规划“多规合一”并联审批，推行工业项目行政审批告知承诺制。完善投融资机制，对市属国有企业进行整合，新建市金融投资有限公司，做实政府融资平台。推进农村“4+2”改革，在沙坡头区开展城乡建设用地增减挂钩改革试点。深化农村集体经济股份制改革和农村承包土地股份合作制改革。培育土地股份制合作社20个，发展土地托管型服务合作社10个。推进城市管理执法体制改革，完善综合执法法律制度。加快事业单位公车改革。推进工业园区电力体制改革，成立中卫、中宁工业园区售电公司，力促重点企业天然气直供。

九、保障改善民生，增进人民群众福祉。办好12件民生实事。突出“有土”“离土”扶贫两篇文章，集中精力抓好产业培育、易地搬迁、整村推进、金融扶持、闽宁协作等重点工作，年内减贫4.6万人，58个重点贫困村脱贫销号。认真落实就业扶持政策，加强就业技能培训，新建创新创业基地（创客空间）5个，确保高校毕业生登记就业率达85%以上。开工建设中卫市慈爱康复中心，推进民办养老机构建设，全市养老服务床位达4285张。持续推进义务教育均衡发展，突出抓好学前教育，新建、改扩建中小学（幼儿园）27所3.3万平方米，改造学校运动场12.3万平方米，海原县义务教育均衡发展通过国家评估认定。完善教育资助体系，保障进城务工人员随迁子女、农村留守儿童等特殊群体受到良好教育。开工建设市人民医院医技楼、市中医医院迁建等项目，建成市疾病预防控制中心业务楼，翻（扩）建镇罗等4个乡镇卫生院门诊楼。深化第四批公立医院国家联系试点城市工作，二级以上医疗机构取消药品加成。狠抓山区、生态移民及流动人口治理，依法打击“两非”行为。建成3个全民健身中心。组织开展品牌文化体育活动，全年演出800场次以上。创排一批优秀文艺精品，培育具有中卫特色的文化品牌。积极申报国家级文化产业示范基地和示范户，加快文化产业发展。办好丝绸之路国际汽车拉力赛等重大赛事。着力化解矛盾纠纷，依法依规解决群众合理诉求。开展公共安全隐患治理，严惩各种违法犯罪活动，营造更加安全、和谐、稳定的发展环境。

各位代表！蓝图催人奋进，圆梦需要实干。让我们更加紧密地团结在以习近平同志为核心的党中央周围，在自治区党委﹑政府和市委的坚强领导下，紧紧依靠全市人民，大力弘扬“不到长城非好汉”的宁夏精神，强化“双争意识”，解放思想、务实苦干，转型追赶、弯道超车，为加快建设开放富裕和谐美丽中卫、全面建成小康社会而努力奋斗！
